# Electrochemical characterisation of new textile electrodes based on a conductive silicon yarn for bioelectrical stimulation

**DOI:** 10.1038/s41598-026-40950-4

**Published:** 2026-03-04

**Authors:** Irene Lange, Tim Kalla, Laureen Wegert, Gerald Rosner, Christoph Müller, Jens Haueisen, Alexander Hunold

**Affiliations:** 1 Institute of Biomedical Engineering and Informatics, Technische Universität Ilmenau, Ilmenau, Germany; 2 neuroConn GmbH, Ilmenau, Germany; 3 warmX GmbH, Apolda, Germany

**Keywords:** Stimulation electrode, Surface electrode, Functional electrical stimulation, Electrochemical impedance spectroscopy, Electrochemical noise, Electrode potential, Chemistry, Energy science and technology, Engineering, Materials science

## Abstract

Transcutaneous electrical stimulation for therapeutic applications relies on electrodes that interface with the human skin. Alongside hydrogel and rubber electrodes, textile electrodes are becoming increasingly important. We introduce a novel textile electrode based on conductive silicone yarn and analyse the electrochemical characteristics. We consider two realisations of the electrode, one with a snap lead button and one where the yarn itself is used for contacting to a cable. Characterisation was done in contact to 0.9% sodium chloride solution. Impedance spectroscopy was performed over 24 h in the frequency range from 0.1 Hz to 1 MHz. Open-circuit potential measurements were taken over 12 h. Voltage and current noise were analysed in the time domain for one hour, and the power spectral density was calculated for a frequency range from 0 to 500 Hz. Our new textile electrodes showed median impedances of 19.6 kΩ at 0.1 Hz and 98 Ω at 1 MHz, which are similar or lower compared to previously introduced stimulation electrodes. The conductive silicone yarn exhibited higher impedances. Noise characteristics and potential stability of the electrodes and the electrode material were comparable to conventional types. In addition to their electrochemical performance, the new textile electrodes offer the skin compatibility of rubber electrodes and the high comfort of textile electrodes, rendering them a promising, sustainable and user-friendly alternative for bioelectrical stimulation applications.

## Introduction

Functional electrical stimulation is a widely used therapeutic method for restoring damaged or lost neurological functions, for example, after a stroke, for rehabilitation after accidents and surgeries, in other cases of nerve damage and in diseases such as multiple sclerosis^1,2^. Electrical stimulation modalities such as functional electrical stimulation, but also transcranial electrical stimulation or peripheral nerve stimulation rely on electrodes interfacing with the human body. Transcutaneous electrical stimulation uses surface electrodes that are applied to the skin above the target tissue.

There are several important requirements for stimulation electrodes^3–5^. They need to be flexible to ensure optimal alignment with the skin curvature. Furthermore, the electrode material must be biocompatible and not cause skin irritation. The electrodes should be easy to handle, and simple self-application would be desirable. Low contact impedance between the electrode and skin is also important, as it ensures efficient current transfer. In addition, electrodes should provide a homogeneous current density distribution across the contact area. This improves stimulation efficiency and increases patient safety by reducing local current density peaks that can cause thermal irritation or skin lesions.

Currently, transcutaneous electrical stimulation commonly uses self-adhesive hydrogel or carbon doped rubber electrodes, typically in combination with a moist sponge pocket^6,7^. A disadvantage of both electrodes is the individual positioning of these for each application, which is not easy to reproduce, time-consuming and limits self-application. Hydrogel electrodes, while easy to apply, may cause skin irritation due to the hydrogel components. In contrast, rubber electrodes—typically rubber or silicone doped with carbon black—have generally a good skin compatibility. However, their application often requires fixation using rubber bands or adhesive tape, which is impractical for regular use.

In addition, textile electrodes are becoming increasingly important in recent applications of transcutaneous bioelectric stimulation^8–10^. They offer many advantages, such as high wearing comfort, reusability, as well as quick and easy reproducible application. There are different methods of integrating conductive electrode material into non-conductive textiles. One option is to cut and sew a conductive fabric onto a textile, another is to apply a conductive paste or ink to a textile. It is also possible to integrate conductive yarn into a textile, for example by embroidering, or to integrate them seamlessly in a single process step, for example by knitting with conductive yarn^11^. Knitted electrodes have the advantage of being stretchable and therefore able to conform well to the skin, even under pressure, to improve electrode–skin contact and comfort^11^. So far, metal-coated yarns, mostly made of silver, have been used for textile electrodes^12–14^. However, silver exhibits antiseptic properties, which increase when an electric current is applied^15^. Carbon black integrated into rubber or silicone is already used as electrode material in textile applicators. In these realizations, the rubber electrodes are either sewn onto the textile or applied afterwards as a conductive paste^16,17^.

In this paper, we present new textile electrodes, which are knitted from carbon doped silicone yarn, representing a new material-structure combination for textile electrodes. They combine the good skin compatibility of conventional rubber electrodes with the high wearing comfort of textile electrodes, making them a promising and user-friendly alternative for bioelectrical stimulation applications. The aim of this paper is to present the electrochemical characteristics of our new textile electrodes. We analyse the impedance spectra, electrode potential and electrochemical noise of the electrode and the electrode material. Two connection variants of the electrode were considered: one with direct metal contact at the electrode implemented by a standard push button for snap lead connection and one where the conductive yarn of the textile material is used for the connection to the cable. These variants are relevant in different use cases for the electrodes, either for classical snap lead connection in clinics/labs or for textile wearables.

## Methods

### Textile electrodes

**Fig. 1:**
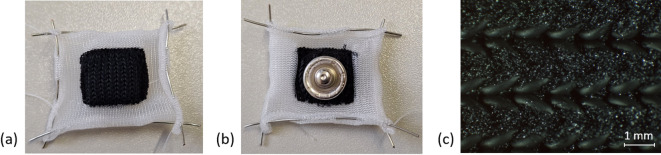
Structure of the textile electrode of conductive silicone yarn. The electrode consists of a 1 × 1 cm^2^ black knitted structure made of conductive silicone yarn, surrounded by a 0.5 cm wide knitted structure made of white non-conductive silicone yarn, which acts as a diffusion barrier. The diffusion barrier is mechanically tensioned and fixed using a wire. (**a**) front of the electrode; (**b**) back of the electrode with attached push button; (**c**) microscopic image of the electrode surface.

The new textile electrodes (Figure 1) are based on a conductive carbon doped silicone yarn. The yarn was machine knitted together with a mechanically stabilising polyamide thread in two layers. A sponge was added to the resulting pocket to implement an electrolyte reservoir. Before each use, the electrode is moistened with 0.9% sodium chloride (NaCl) solution, which is filled in the electrolyte reservoir. The push button and the electrolyte reservoir are not insulated from each other and are in direct contact.

A diffusion barrier made of non-conductive silicone yarn was knitted around the electrode. This barrier can prevent diffusion of the electrolyte into the non-conductive textile when the electrode is integrated into a textile applicator. A push button on the back of the electrode can connect to a stimulator’s electrode cable.

The textile electrodes can be made in various shapes and sizes. For use in bioelectrical stimulation, they can be integrated into a textile applicator for simplified usability.

### Measurement setup

The electrochemical characterisation of the textile electrodes comprised electrochemical impedance spectroscopy (EIS), open circuit potential (OCP), and electrochemical noise (ECN) measurements. We used a Reference 600 + (Gamry Instruments, USA) to perform all electrochemical characterisation measurements of the textile electrodes at room temperature between 21 and 25 °C. The setup under test was placed in a Faraday cage to minimise external interference.

The electrode material itself and the new textile electrode, with its push button connector, were characterised electrochemically. Textile samples measuring 1 × 1 cm^2^ were used to characterise the electrode with the push button, as described in the section Textile electrodes. For the electrode material characterisation, samples of the same size were used, but without a push button, because this additional material potentially influences the electrochemical properties of the electrode. The silicone yarn used in the electrode was sewn to the back of the electrode for contacting the electrodes to the measurement setup. Two practical connection variants were considered. In the first variant, the contact to the cable connecting to the Reference 600 + was implemented directly at the electrode with the push button. Both the yarn and the push button were in direct contact with the electrolyte. In the second variant, the contact to the cable was positioned slightly away from the electrode via the conductive yarn, and that contact was isolated from the electrolyte. This second variant also serves for the material characterisation of the conductive textile yarn, as only the yarn was in contact with the electrolyte. Both connection variants represent realistic and relevant application scenarios for textile stimulation electrodes. In the following, the sample electrodes are termed electrodes with button, the samples for material characterisation are termed electrodes without button.

**Fig. 2:**
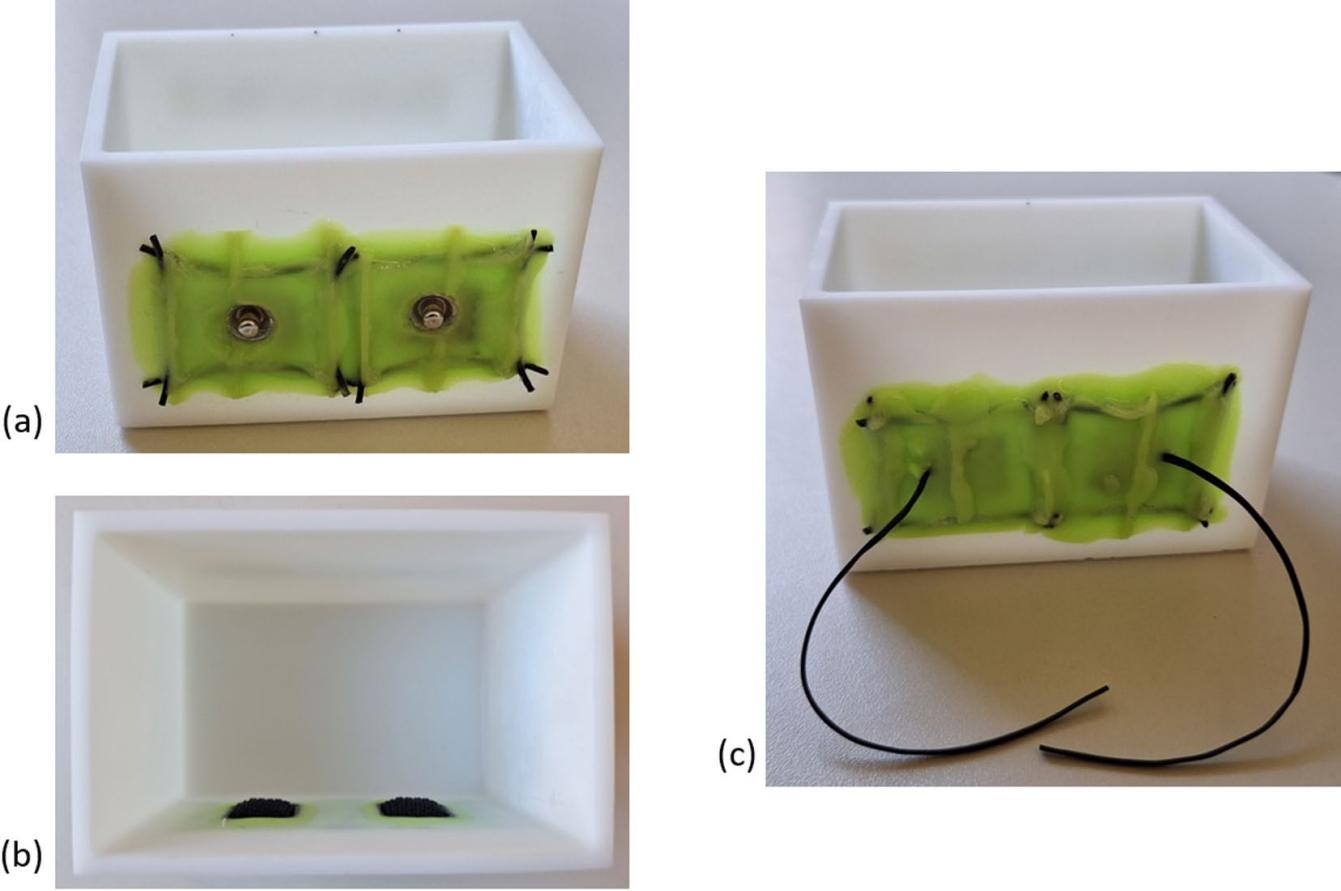
3D-printed electrode box measuring 9 cm × 6 cm × 6 cm with integrated 1 × 1 cm^2^ electrodes placed 2 cm apart. The electrodes are stretched and fixed to the box with silicone to ensure a defined interface between the electrodes and the electrolyte: (**a**) front view of the box with electrodes with button; (**b**) interior view of the box with electrodes with button; (**c**) front view of the box with electrodes without button.

All characterisation measurements were performed in 0.9% NaCl solution. To ensure a controlled and consistent interface between the electrodes and the electrolyte, a box was designed with Autodesk Inventor Professional 2023 (Autodesk GmbH, Germany) and 3D printed using standard resin (Anycubic Technology Co., Ltd., China) with the Anycubic Photon Mono X (Anycubic Technology Co., Ltd., China). The box is 9 cm × 6 cm × 6 cm in size and has two holes on one side for attaching two 1 × 1 cm^2^ electrodes, placed 2 cm apart edge to edge. The electrodes were watertight attached to the electrode box using silicone. The box for the electrode characterisation is shown in Fig. 2. We manufactured two electrode boxes, one for the electrodes with button and one for the electrodes without button.

The NaCl solution was degassed to prevent the formation of air bubbles on the electrodes over the measurement period to avoid interaction with the measurement. After filling the electrode box with NaCl solution, potentially forming air bubbles on the textile electrodes were carefully removed.

In the present measurement setup, the electrode was in contact with aqueous electrolyte solution. Thus, the electrolyte solution diffused into the knitted textile and fully immersed the conductive thread without the application of contact pressure.

### Electrochemical impedance spectroscopy

Potentiostatic two-electrode EIS was performed in the frequency range from 0.1 Hz to 1 MHz, recording ten samples per decade. EIS on one pair of electrode samples was repeated every hour for 24 h. Such a series of measurements was performed three times for one pair of electrodes with button and one pair of electrodes without button. Each series of measurements was performed with new NaCl solution. The electrodes and boxes were washed and completely dried in between. The left electrode was always used as working electrode and the right electrode as counter and quasi-reference electrode.

The repeatedly measured impedance amplitudes and phases were tested for normal distribution using the Lilliefors test^18^, separately for each electrode type, with and without button, at each frequency from 25 runs in each three series.

### Electrode potential

The OCP measurements were taken over a period of 12 h, with the OCP being sampled every second. The OCP was measured three times for each of the two electrodes with button and two electrodes without button.

The reference electrode was a sintered silver/silver-chloride (Ag/AgCl) electrode (MedCaT BV, Netherlands) with a diameter of 12 mm. The reference electrode was placed on the opposite wall, opposite to the measuring electrode in the electrode box. The reference electrode and the measuring electrode were 5 cm apart.

The OCP is the potential difference between the reference electrode and the measuring electrode. The Ag/AgCl electrode has an electrode potential of 222 mV^19^. The electrode potential of the textile electrode is calculated using the following equation:

1 $$E_{Test} = OCP + E_{Ref}$$

$E_{Test}$ is the electrode potential of the electrode under test, $E_{Ref}$ is the electrode potential of the reference electrode and $OCP$ is the measured potential difference between the two electrodes.

New NaCl solution was used for each measurement. The electrodes and boxes were washed and completely dried between the measurements.

### Electrochemical noise

The ECN was recorded using the Zero Resistance Ammeter (ZRA) mode to record current and voltage noise simultaneously. Again, a sintered Ag/AgCl electrode (MedCaT BV, Netherlands) with a diameter of 12 mm was used as the reference electrode. The reference electrode was positioned in the centre, opposite the textile electrodes, inside the box. The left textile electrode was used as the working electrode and the right textile electrode as the counter electrode. All measurements were performed using one pair of electrodes with button and one pair of electrodes without button.

To analyse the electrochemical noise in the time domain, measurements were taken for one hour. The root mean square (RMS) of the current and voltage noise was determined and stored every three seconds based on 1000 samples from the last second. The current and voltage noise for the electrodes with and without button were tested for normal distribution using the Lilliefors test^18^.

For the analysis in the frequency domain, 1000 samples per second were recorded over 10 s. This measurement was performed twice, immediately before and after the one hour measurement for analysis of the voltage and current noise in the time domain. The same measurement was repeated on three consecutive days. Further, reference measurements were also taken. For the reference measurements, all channels were short-circuited using a wire. The power spectral density (PSD) was calculated for the current and voltage noise in a frequency range from 0 to 500 Hz in intervals of 0.1 Hz. The PSD for current and voltage noise for the electrodes with and without button were tested for normal distribution using the Lilliefors test^18^.

The electrodes and boxes were washed and completely dried between the measurement days. New NaCl solution was used for each day of measurements.

## Results

### Impedance spectroscopy

**Fig. 3:**
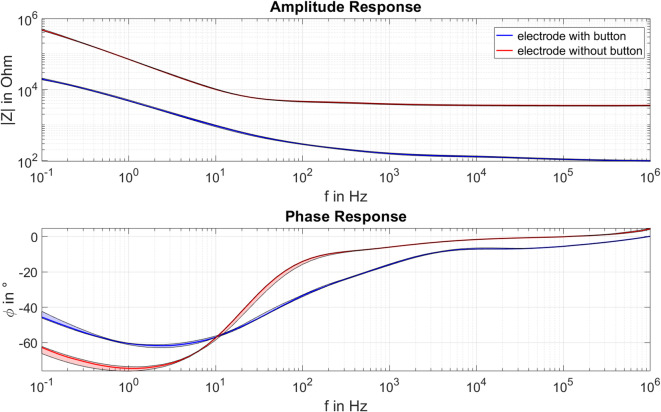
The median and the 25th and 75th percentiles (shaded areas) of the impedance amplitude (top) and phase (bottom) for the textile electrodes with (blue) and without (red) button.

According to the Lilliefors test, normal distribution of impedance amplitude and phase could not be assumed at each frequency for both electrode types. Therefore, the medians and the 25th and 75th percentiles were calculated for the measured amplitudes and phases to analyse the data for the electrodes with and without button, per frequency. Figure 3 presents the median, as well as the 25th and 75th percentiles, of the impedance amplitude and phase spectra for both electrode types.

It is evident that the electrode without button has a much higher impedance than the electrode with button over the entire tested frequency range. The electrode with button also has a smaller extreme value in the phase spectrum.

For the electrode with button, the median impedance is 19.6 kΩ at 0.1 Hz and decreases to 98 Ω at 1 MHz, with a minimum phase of -61.8 degrees observed at 2.5 Hz. In contrast, the electrode without button shows a median impedance of 469.7 kΩ at 0.1 Hz and 3.6 kΩ at 1 MHz, with a minimum phase of -74.6 degrees occurring at 1.3 Hz.

The variability in amplitude and phase response over 24 h was calculated based on medians and percentiles. The maximum variability of the impedance amplitude is comparable for both electrode types, with 7.1% at 1.3 Hz for the electrodes with button and 7.2% at 0.1 Hz for those without button. The impedance phase shows a lower maximum variability of 5.5% at 0.1 Hz for the electrode without button than for the electrode with button, which has a maximum variability of 7.1% at 0.1 Hz.

### Electrode potential

**Fig. 4:**
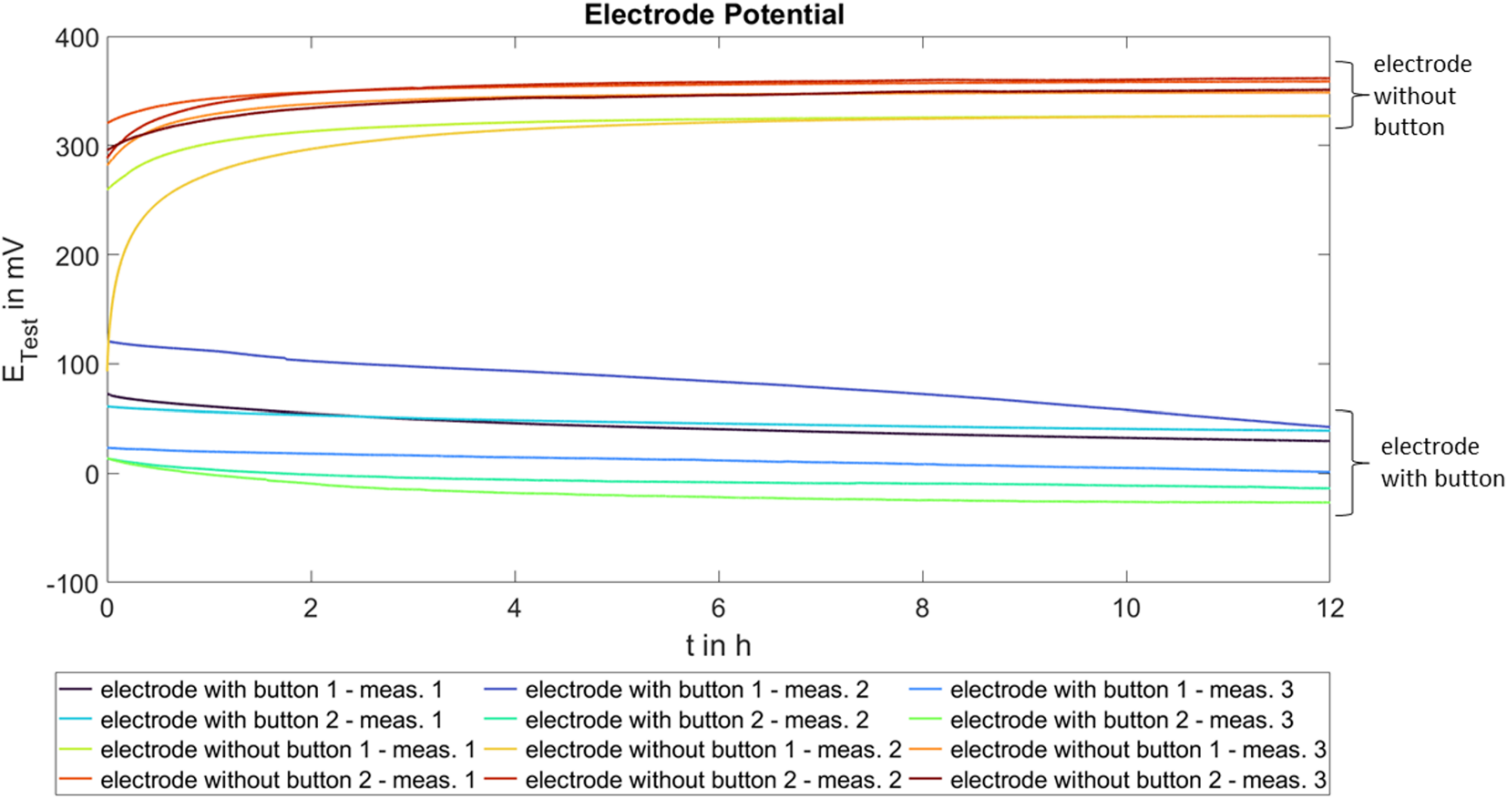
Electrode potentials of the textile electrodes with and without button, three measurements (meas.) per electrode.

Figure 4 shows the electrode potentials of the two electrodes with button and the two electrodes without button. Three measurements were taken per electrode over 12 h.

The two electrode types show clearly different electrode potentials. It is evident that the electrode potentials are more reproducible and stable for the electrodes without button. At the beginning of the measurement, a clear increase in potential is visible, which stabilises after a few hours. For the electrodes with button, a higher variability in the potential curves is observed. The median electrode potential for the electrodes without button is approximately 349.8 mV after 12 h, with the 25th and 75th percentiles ranging from 327.1 to 358.7 mV. In contrast, the median electrode potential for the electrodes with button is 15.4 mV after 12 h, with the corresponding 25th and 75th percentiles between –13.7 to 39.1 mV.

### Electrochemical noise

**Fig. 5:**
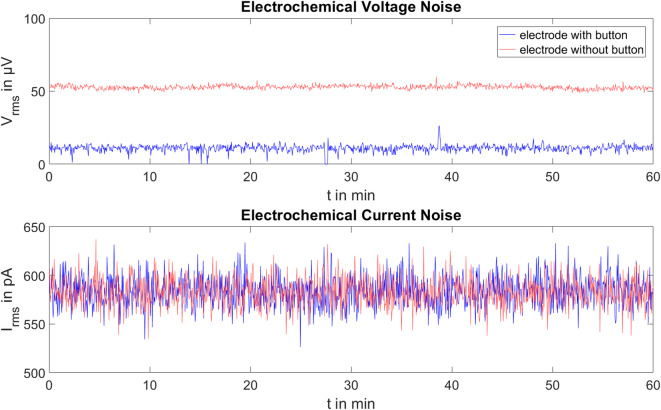
Root mean square values of the electrochemical voltage and current noise for one pair of electrodes with button (blue) and one pair of electrodes without button (red) over one hour. Median voltage noise is 11 µV for electrodes with button and 53 µV for electrodes without button, while median current noise is comparable at 583 pA and 582 pA.

Figure 5 shows the voltage and current noise of the two electrode types in the time domain over one hour.

As the Lilliefors test did not detect a normal distribution of current and voltage noise for the electrodes with and without button, the evaluation is based on the median values. For the electrodes with button, the median voltage noise is 11 µV, and the median current noise is 583 pA. The median current noise of the electrodes without button is comparable to that of the electrodes with button at 582 pA. However, the voltage noise of the electrodes without button is higher, at 53 µV.

**Fig. 6:**
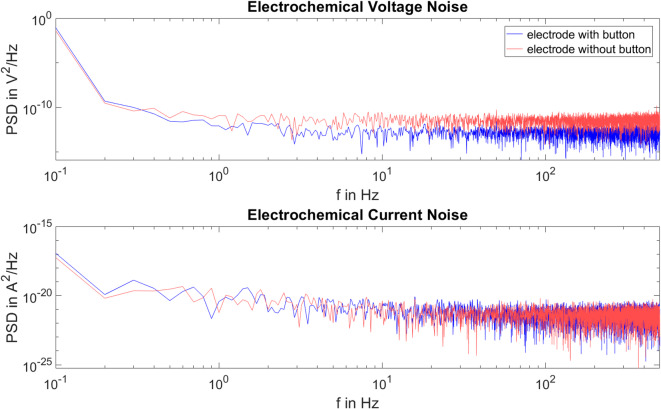
Power spectral density of the voltage and current noise of the measurement before time domain measurement for one pair of electrodes with button (blue) and one pair of electrodes without button (red) in the frequency range from 0 to 500 Hz.

Figure 6 shows the PSD of the voltage and current noise for the two electrode types. The electrochemical noise measurement used to analyse the PSD was recorded immediately before the time domain analysis measurement of the current and voltage noise.

As the Lilliefors test shows that the PSD of voltage and current noise is not normally distributed, further evaluation is based on the medians.

For the electrodes with button, the measurement shown in Fig. 6 results in a median voltage noise power of 1.65 × 10^–13^ V^2^/Hz and a median current noise power of 4.57 × 10^–22^ A^2^/Hz. The electrodes without button show comparable current noise power with a median of 4.43 × 10^–22^ A^2^/Hz, but show higher voltage noise power with a median of 3.77 × 10^–12^ V^2^/Hz.

The noise measurement performed immediately after the one hour measurement for time domain analysis provides comparable results for the electrodes with and without button. For electrodes with button, the median voltage noise power is 1.82 × 10^–13^ V^2^/Hz, while the median current noise power is 4.39 × 10^–22^ A^2^/Hz. The electrodes without button show a voltage noise power of 3.86 × 10^–12^ V^2^/Hz and a current noise power with a median of 4.63 × 10^–22^ A^2^/Hz.

**Fig. 7:**
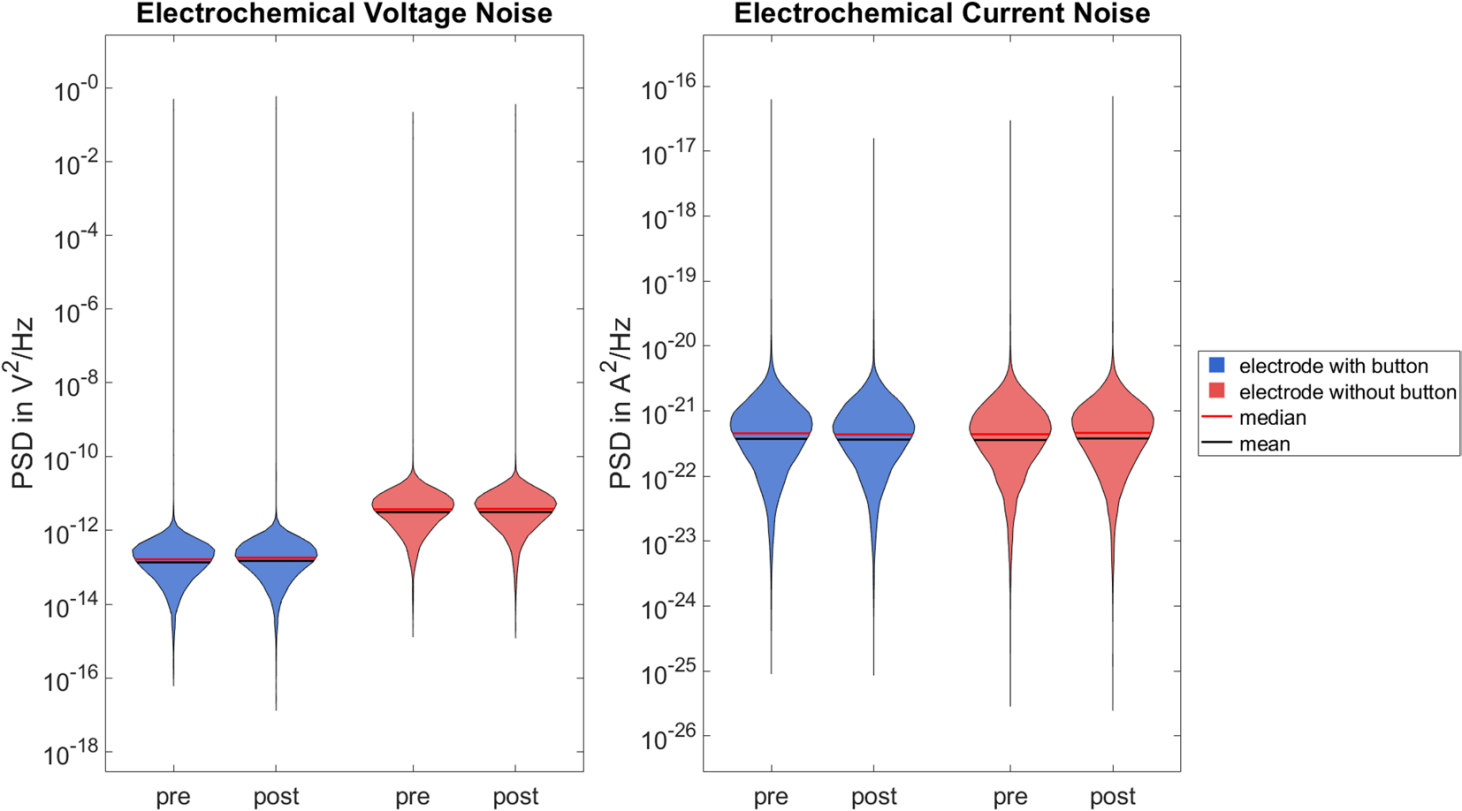
Violin plots showing the distribution of the power spectral density of voltage (left) and current (right) noise for one pair of the electrodes with (blue) and without (red) button of the noise measurement immediately before (pre) and after (post) the measurement for time domain analysis in the frequency range from 0 to 500 Hz. Median voltage and current noise powers are: with button—1.65 × 10^–13^ V^2^/Hz and 4.57 × 10^–22^ A^2^/Hz (pre), 1.82 × 10^–13^ V^2^/Hz and 4.39 × 10^–22^ A^2^/Hz (post); without button—3.77 × 10^–12^ V^2^/Hz and 4.43 × 10^–22^ A^2^/Hz (pre), 3.86 × 10^–12^ V^2^/Hz and 4.63 × 10^–22^ A^2^/Hz (post).

Figure 7 shows the PSD of voltage and current noise measured immediately before and after the noise measurement for time domain analysis. Violin plots are used to compare the electrodes with and without button.

The three additional measurement days also show reproducible results with consistent median values for voltage and current noise power. For the electrodes with button, the median PSD for voltage noise on day one is 1.88 × 10^–13^ V^2^/Hz, on day two it is 1.80 × 10^–13^ V^2^/Hz and on day three it is 1.72 × 10^–13^ V^2^/Hz. The corresponding PSD values for current noise are 4.45 × 10^–22^ A^2^/Hz on day one, 4.65 × 10^–22^ A^2^/Hz on day two, and 4.49 × 10^–22^ A^2^/Hz on day three. The electrodes without button show a median PSD for voltage noise of 5.56 × 10^–12^ V^2^/Hz on the first day, 2.86 × 10^–12^ V^2^/Hz on the second day and 5.51 × 10^–12^ V^2^/Hz on the third day. The median PSD of the current noise is 4.58 × 10^–22^ A^2^/Hz on the first day, 4.40 × 10^–22^ A^2^/Hz on the second day and 4.55 × 10^–22^ A^2^/Hz on the third day.

Overall, the noise behaviour before and after the one hour time domain analysis measurement, and over several days, is very consistent.

The reference measurements on the three measurement days showed medians of 1.30 × 10^–13^ V^2^/Hz, 1.23 × 10^–13^ V^2^/Hz and 1.26 × 10^–13^ V^2^/Hz for the PSD of the voltage noise and 3.00 × 10^–20^ A^2^/Hz, 3.01 × 10^–20^ A^2^/Hz and 2.66 × 10^–20^ A^2^/Hz for the PSD of the current noise. Compared to our electrodes, the voltage noise has a similar median to that of the electrodes with button, while the current noise is much higher than for both electrode types.

**Fig. 8:**
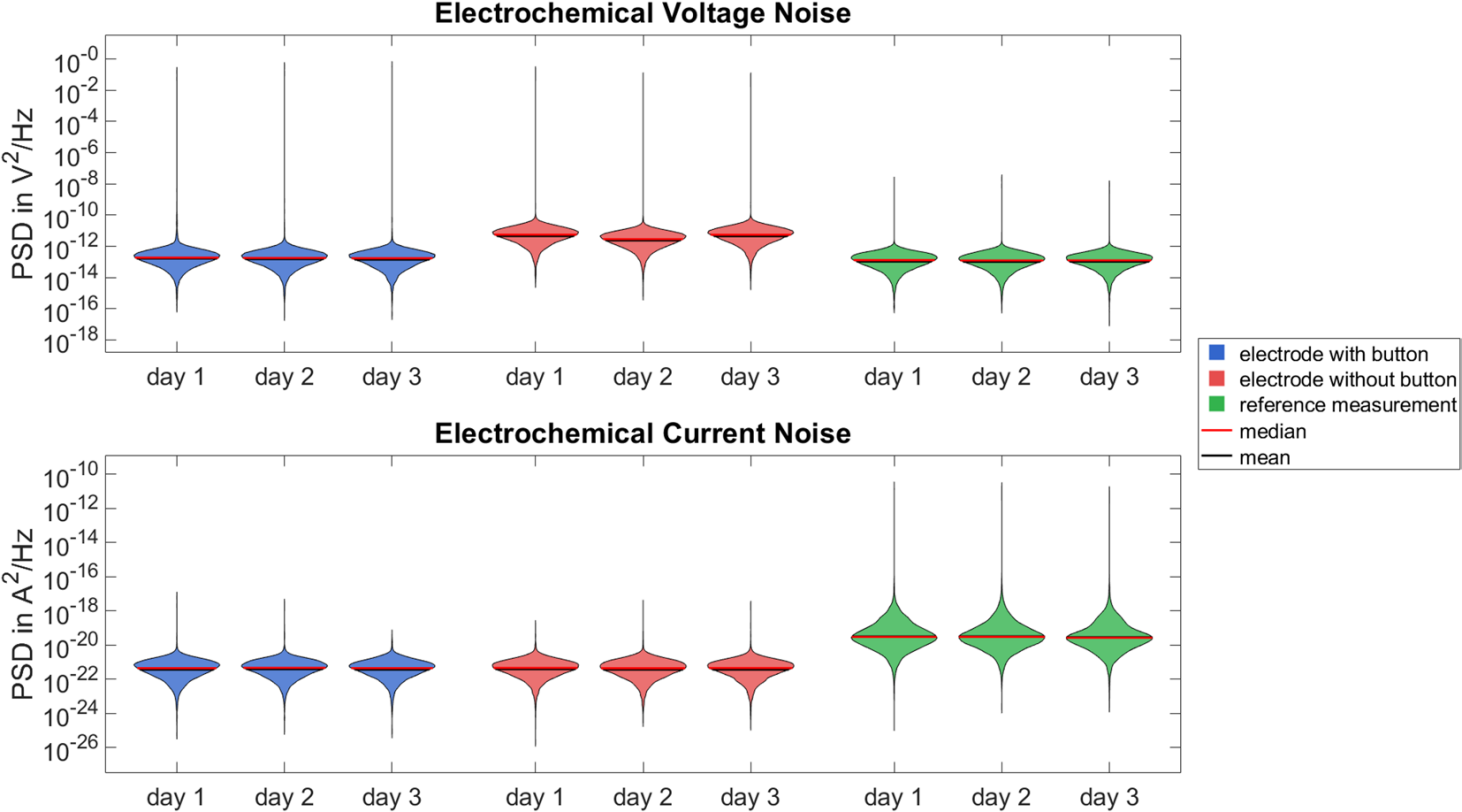
Violin plots showing the distribution of the power spectral density of voltage and current noise for one pair of the electrodes with button (blue), one pair of the electrodes without button (red), and the reference (green) measurements over the three additional measurement days in the frequency range from 0 to 500 Hz. Median voltage and current noise powers are: with button—voltage: 1.88 × 10^–13^ V^2^/Hz, 1.80 × 10^–13^ V^2^/Hz, 1.72 × 10^–13^ V^2^/Hz; current: 4.45 × 10^–22^ A^2^/Hz, 4.65 × 10^–22^ A^2^/Hz, 4.49 × 10^–22^ A^2^/Hz; without button—voltage: 5.56 × 10^–12^ V^2^/Hz, 2.86 × 10^–12^ V^2^/Hz, 5.51 × 10^–12^ V^2^/Hz; current: 4.58 × 10^–22^ A^2^/Hz, 4.40 × 10^–22^ A^2^/Hz, 4.55 × 10^–22^ A^2^/Hz; reference—voltage: 1.30 × 10^–13^ V^2^/Hz, 1.23 × 10^–13^ V^2^/Hz, 1.26 × 10^–13^ V^2^/Hz; current: 3.00 × 10^–20^ A^2^/Hz, 3.01 × 10^–20^ A^2^/Hz, 2.66 × 10^–20^ A^2^/Hz.

Figure 8 shows the PSD of the voltage and current noise over the three additional measurement days, comparing the electrodes with and without button and the reference measurements using violin plots.

Table 1 summarises the PSD values for voltage and current noise, showing the median, 25th and 75th percentiles, as well as the minimum and maximum values. This provides an overview of the noise distribution for electrodes with and without button across pre- and post-measurements, and on multiple measurement days.

**Table 1:** Median, 25th percentile, 75th percentile, minimum, and maximum values of the power spectral density of voltage and current noise for one pair of electrodes with and without button before and after time-domain measurements and over multiple measurement days in the frequency range from 0 to 500 Hz.

			Median	25th percentile	75th percentile	Minimum	Maximum
Electrode with button	Pre	PSD in V^2^/Hz	1.65 × 10^–13^	6.75 × 10^–14^	3.35 × 10^–13^	1.18 × 10^–16^	0.25
		PSD in A^2^/Hz	4.57 × 10^–22^	1.87 × 10^–22^	9.17 × 10^–22^	1.74 × 10^–25^	3.24 × 10^–17^
	Post	PSD in V^2^/Hz	1.82 × 10^–13^	7.46 × 10^–14^	3.66 × 10^–13^	2.50 × 10^–17^	0.31
		PSD in A^2^/Hz	4.39 × 10^–22^	1.88 × 10^–22^	8.68 × 10^–22^	1.64 × 10^–25^	8.20 × 10^–18^
Electrode without button	Pre	PSD in V^2^/Hz	3.77 × 10^–12^	1.55 × 10^–12^	7.63 × 10^–12^	2.48 × 10^–15^	0.12
		PSD in A^2^/Hz	4.43 × 10^–22^	1.85 × 10^–22^	8.81 × 10^–22^	5.45 × 10^–26^	1.55 × 10^–17^
	Post	PSD in V^2^/Hz	3.86 × 10^–12^	1.61 × 10^–12^	7.60 × 10^–12^	2.27 × 10^–15^	0.19
		PSD in A^2^/Hz	4.63 × 10^–22^	1.90 × 10^–22^	9.24 × 10^–22^	4.73 × 10^–26^	3.63 × 10^–17^
Electrode with button	Day 1	PSD in V^2^/Hz	1.88 × 10^–13^	7.77 × 10^–14^	3.86 × 10^–13^	1.16 × 10^–16^	0.15
		PSD in A^2^/Hz	4.45 × 10^–22^	1.74 × 10^–22^	9.22 × 10^–22^	6.12 × 10^–26^	6.46 × 10^–18^
	Day 2	PSD in V^2^/Hz	1.80 × 10^–13^	7.49 × 10^–14^	3.65 × 10^–13^	3.30 × 10^–17^	0.31
		PSD in A^2^/Hz	4.65 × 10^–22^	1.94 × 10^–22^	9.36 × 10^–22^	1.10 × 10^–25^	2.62 × 10^–18^
	Day 3	PSD in V^2^/Hz	1.72 × 10^–13^	6.70 × 10^–14^	3.47 × 10^–13^	3.83 × 10^–17^	0.36
		PSD in A^2^/Hz	4.49 × 10^–22^	1.85 × 10^–22^	8.86 × 10^–22^	6.79 × 10^–26^	4.12 × 10^–20^
Electrode without button	Day 1	PSD in V^2^/Hz	5.56 × 10^–12^	2.32 × 10^–12^	1.09 × 10^–11^	4.25 × 10^–15^	0.18
		PSD in A^2^/Hz	4.58 × 10^–22^	1.94 × 10^–22^	9.15 × 10^–22^	2.18 × 10^–26^	1.51 × 10^–19^
	Day 2	PSD in V^2^/Hz	2.86 × 10^–12^	1.18 × 10^–12^	5.80 × 10^–12^	6.72 × 10^–16^	0.07
		PSD in A^2^/Hz	4.40 × 10^–22^	1.79 × 10^–22^	9.12 × 10^–22^	3.24 × 10^–25^	2.21 × 10^–18^
	Day 3	PSD in V^2^/Hz	5.51 × 10^–12^	2.27 × 10^–12^	1.10 × 10^–11^	3.04 × 10^–15^	0.07
		PSD in A^2^/Hz	4.55 × 10^–22^	1.83 × 10^–22^	8.96 × 10^–22^	1.95 × 10^–25^	1.99 × 10^–18^
Reference meas.	Day 1	PSD in V^2^/Hz	1.30 × 10^–13^	5.28 × 10^–14^	2.63 × 10^–13^	1.02 × 10^–16^	1.43 × 10^–8^
		PSD in A^2^/Hz	3.00 × 10^–20^	1.02 × 10^–20^	9.67 × 10^–20^	2.52 × 10^–25^	1.39 × 10^–11^
	Day 2	PSD in V^2^/Hz	1.23 × 10^–13^	5.14 × 10^–14^	2.49 × 10^–13^	9.99 × 10^–17^	1.99 × 10^–8^
		PSD in A^2^/Hz	3.01 × 10^–20^	1.06 × 10^–20^	9.21 × 10^–20^	2.58 × 10^–24^	1.32 × 10^–11^
	Day 3	PSD in V^2^/Hz	1.26 × 10^–13^	5.30 × 10^–14^	2.51 × 10^–13^	1.46 × 10^–17^	8.48 × 10^–9^
		PSD in A^2^/Hz	2.66 × 10^–20^	9.46 × 10^–21^	8.32 × 10^–20^	2.97 × 10^–24^	7.67 × 10^–12^

## Discussion

Electrochemical characterisation is an essential part in the evaluation of new electrodes. It allows the investigation of the impedance, electrode potential and electrochemical noise of different materials.

The impedance is one of the most important characteristics of an electrode. The impedance is primarily characterised by the contact impedance between the electrode and the electrolyte, especially in the low frequency range. The contact impedance is influenced by various parameters such as the electrode material, the contact pressure, the electrode size or the surface texture of the electrode^13^. The electrode potential and electrochemical noise influence signal quality and are particularly important when recording bioelectrical signals. A stable and fast-setting electrode potential and low electrochemical noise are important to ensure that the low amplitudes of the biosignals are not overlaid and distorted. This also ensures a stable load on the current source during bioelectrical stimulation.

Due to their reliable performance, Ag/AgCl electrodes are commonly used as reference electrodes for electrochemical characterisation and as standard electrodes for recording bioelectrical signals. In 0.9% NaCl solution, Ag/AgCl electrodes exhibit a stable and low impedance of about 10 Ω to 20 Ω over a frequency range of 10 Hz to 10 kHz^20^. The Ag/AgCl electrode has a stable electrode potential of 222 mV^19,20^ and also shows low noise, with an RMS voltage noise value of 3.5 µV^21^.

The impedance characteristics of our two electrode types show clear differences. The electrodes with button show considerably lower impedances over the whole frequency range. The only difference between the two types of electrodes is in their contacts. The complete electrodes have a push button for contact, while the electrodes used for material characterisation have an additional conductive silicone yarn attached for contact. This yarn, which is made of the same material as the electrodes, is 10 cm long. This adds a total of 20 cm of yarn with a resistance of approximately 2.8 kΩ to the test setup, containing two electrodes. This results in an impedance offset between the electrodes with button and the electrodes without button. Even after subtracting this offset, the impedance of the electrode without button is higher over the entire frequency range, especially at low frequencies. Further, the electrodes without button show a stronger dependence of the impedance on the frequency. The push button, with its high conductivity compared to the yarn, reduces the impedance of the electrode by providing an additional metal connection. The low variability in the impedance of the electrodes with and without button indicates stable performance over time and multiple measurements. This is especially important for long-term applications in order to ensure reliability and reproducibility.

Since the push button for the electrode is important for connecting to the stimulation setup in later applications, we will compare the results of the complete electrode with other electrodes known from the literature.

Zhou et al.^14^ characterised new textile electrodes in dry and wet conditions with hydrogel electrodes on the leg. The wet textile electrodes showed the lowest impedance over a frequency range from 0.1 Hz to 100 kHz. In comparison, our electrodes show considerably lower impedances in the low frequency range and comparable impedances in the high frequency range. At 1 kHz, the impedance of the textile electrode in Zhou et al. was 950 Ω, while our electrode have an impedance of only 160 Ω. Hunold et al.^12^ characterised new textile electrodes in a sham stimulation protocol at 85 Hz on the head for transcranial electrical stimulation and compared them with rubber electrodes placed in a moistened sponge pocket. The average impedance values were 10 kΩ for the textile electrodes and 5.3 kΩ for the rubber electrodes. In comparison, our electrodes show lower impedances around 300 Ω at 85 Hz. Euler et al.^13^ characterised various textile electrodes on the forearm. For moistened electrodes, impedances between 9.6 and 15.8 kΩ were measured at 39 Hz. Our electrodes show 430 Ω for 39 Hz. All three studies used electrodes made of silver-coated yarn. It should be noted that the measurement setups differ very much: while the characterisation was done on test subjects in the literature, our measurements were done in NaCl solution. In summary, compared to the textile electrodes described, our electrodes show much lower impedances, especially in the low frequency range, and less frequency dependence of the impedance.

The electrode potential shows clear differences between our two electrode types. The electrodes without button, which are used for material characterisation, show an electrode potential of approximately 350 mV after the potential settled. In comparison, electrodes with button show a much lower potential of approximately 15 mV after 12 h. This can be explained by the material composition of the push button. The push button is made of stainless steel, which is composed of elements including iron, chromium and nickel. The standard electrode potentials of the elements are -440 mV (Fe), -740 mV (Cr) and -236 mV (Ni)^22^, which are significantly lower than the potential of the electrode material, causing a shift in the electrode potential towards negative values. The higher variability of the electrode potentials of the electrode with button compared to the electrodes without button shows that the push button increases the variability of the electrode potentials.

Fiedler et al.^20^ characterised the electrode potential of different electrode materials. The standard deviation of the Ag/AgCl electrodes was approximately 2 mV while other materials showed relatively unstable potentials with a standard deviation of up to 440 mV. With a maximum difference between individual potential measurements of 113 mV for the electrodes with button, our electrode still offers a relatively stable electrode potential compared to other electrode materials.

When analysing the electrochemical noise, it can be seen that the current noise between the electrodes with and without button shows comparable noise levels. However, in terms of voltage noise, the electrodes without button show a higher noise level in the time domain and the PSD. This indicates that the push button has a positive impact on the voltage noise behaviour. This is caused by the additional conduction path created by the push button, which has a lower impedance than the conductive silicone yarn. The push button made of stainless steel generates less voltage noise than the electrode material^23^. This reduces the voltage noise level of the electrode with button. The reference measurements were recorded to capture the systems’ noise level. These measurements showed that the PSD of the voltage noise is comparable to that of our electrodes with button. In contrast, the PSD of the current noise is higher compared to our electrode types, which is caused by the high conductivity of the wire. This indicates that the voltage noise power of our electrodes with button is primarily determined by system noise.

Compared to Ag/AgCl electrodes^21^, our textile electrodes have a slightly higher voltage noise in the time and frequency domain. Mansfeld et al.^23^ analysed the noise behaviour of a stainless steel electrode. In comparison, the PSD of the current noise is lower for our two electrode types and slightly higher for the PSD of the voltage noise. Maji et al.^24^ characterised various textile dry ECG electrodes and compared them with a silicone rubber electrode and a conventional self-adhesive foam electrode. All electrodes exhibit voltage noise in the frequency domain in the range of nV/√Hz and thus show a slightly lower noise level than our electrodes types.

While not in the primary scope of the development of our electrodes, one may also use them to measure electrophysiological signals such as the electrocardiogram (ECG). The ECG has a relevant signal amplitude of about 10 μV to 5 mV. These biosignal dynamics are higher than the measured noise of our new textile electrodes, consequently these can also be used to record bioelectrical signals such as the ECG.

A limitation of our study is that in our experimental setup, the push button of the complete electrode is in direct contact with the NaCl solution. This means that the electrolyte comes into contact with both the push button and the actual electrode material, the conductive silicone yarn. However, this situation represents the practical application scenario. Additionally, all measurements were performed under static conditions without applying contact pressure or simulating electrode–skin contact. Future work should consider factors such as skin–electrode impedance, pressure variation, movement and long-term application.

Furthermore, only two connection configurations were investigated: direct contact at the electrode, where the push button was in direct contact with the electrolyte, and a connection positioned slightly away from the electrode via the conductive yarn, thereby avoiding direct electrolyte contact. Both configurations are considered relevant for practical and clinical applications. There are a variety of alternative contacting strategies and connection geometries that may influence the electrochemical characteristics of the electrodes and should be investigated in future work.

## Conclusion

Our new textile electrodes present suitable impedance spectra for broad use in bioelectrical applications. Compared to previously introduced textile stimulation electrodes, our new textile electrodes showed comparable or lower impedances. The relatively small variability in amplitude and phase response indicated a stable performance of our new textile electrodes. Although some temporal fluctuations in the electrode potential are observed, they remain within the range typical for established electrode materials. In terms of noise performance, our electrodes also demonstrate values comparable to those of conventional electrode types.

Overall, the new textile electrodes exhibit promising electrochemical properties and represent an alternative to existing technologies in bioelectrical stimulation. They combine the skin compatibility of rubber electrodes with the high comfort of textile electrodes. Their washable and reusable nature contributes to sustainability and cost-efficiency, and integrating them into a textile applicator enables quick and reproducible application. These characteristics highlight the potential of the electrodes for practical, and user-friendly applications in bioelectrical stimulation.

For a conclusive assessment, it is necessary to consider not only the electrochemical characteristics, but also the mechanical characteristics, the integration into a textile applicator, and studies involving volunteers and patients. In future work, we will focus on these aspects to further evaluate the practical applicability of the electrodes. This includes user-centric evaluations, such as usability tests and assessments of skin-contact comfort. Furthermore, electrode–skin contact and mechanical conformity can be investigated using skin phantoms before subsequent electrical stimulation measurements are conducted with volunteers and patients.

## Data Availability

The data are available upon reasonable request to the corresponding author.
